# Assessing the influence of the built environment on physical activity for utility and recreation in suburban metro Vancouver

**DOI:** 10.1186/1471-2458-11-959

**Published:** 2011-12-30

**Authors:** Lisa Oliver, Nadine Schuurman, Alexander Hall, Michael Hayes

**Affiliations:** 1 Health Analysis Division, Statistics Canada, Ottawa, ON, Canada; 2 Department of Geography, Simon Fraser University, British Columbia, Canada; 3 Health Education and Research, University of Victoria, British Columbia, Canada

## Abstract

**Background:**

Physical inactivity and associated co-morbidities such as obesity and cardiovascular disease are estimated to have large societal costs. There is increasing interest in examining the role of the built environment in shaping patterns of physical activity. However, few studies have: (1) simultaneously examined physical activity for leisure and utility; (2) selected study areas with a range of built environment characteristics; and (3) assessed the built environment using high-resolution land use data.

**Methods:**

Data on individuals used for this study are from a survey of 1602 adults in selected sites across suburban Metro Vancouver. Four types of physical activity were assessed: walking to work/school, walking for errands, walking for leisure and moderate physical activity for exercise. The built environment was assessed by constructing one-kilometre road network buffers around each respondent's postal code. Measures of the built environment include terciles of recreational and park land, residential land, institutional land, commercial land and land use mix.

**Results:**

Logistic regression analyses showed that walking to work/school and moderate physical activity were not associated with any built environment measure. Living in areas with lower land use mix, lower commercial and lower recreational land increased the odds of low levels of walking for errands. Individuals living in the lower third of land use mix and institutional land were more likely to report low levels of walking for leisure.

**Conclusions:**

These results suggest that walking for errands and leisure have a greater association with the built environment than other dimensions of physical activity.

## Background

Physical inactivity and associated co-morbidities such as obesity, type 2 diabetes and cardiovascular disease are estimated to have high economic and social costs. Increasing physical activity is considered important to improve public health [1]. While most research on the determinants of physical activity focus on individual factors, there is increasing recognition that patterns of physical activity are also shaped by the contexts in which individuals live. Studies have shown that the social and built characteristics of places individuals reside can either promote or inhibit opportunities for physical activity [2-11].

Relationships between the built environment and physical activity are not well understood and results are often not consistent across studies [12-16]. Several studies have found that individuals living in areas that have high residential density, land use mix, and street connectivity (i.e. neighbourhoods with high 'walkability') have increased levels of physical activity [6,17-25] but consistent relationships between each of these variables and physical activity have not been found across all studies [15,16,26-29]. Similarly, many studies have found that increased access to green space increases physical activity [30-35] while others have not found significant relationships [26,36].

Physical activity for utilitarian purposes, such as commuting to work or school or for errands such as grocery shopping, may be related more closely to different aspects of the built environment than physical activity for leisure and recreation. Multiple studies have shown clear positive associations between high land use mix and walking for transport [25,26,37], however associations with walking for leisure are more uncertain [26,37]. Another measure of the built environment, residential density, has also been associated with increased levels of walking for both leisure and travel [25,27,38] but not for all populations [19]. For example, one study found no associations with any measure of the physical environment in their study of physical activity amongst the elderly [19].

Inconsistent results from different studies may be due to two factors. First, until recently studies relied on perceived measures of the built environment which can have limitations compared to objective measures [16,39,40]. Developments in Geographic Information Systems (GIS) and increasing availability of high resolution spatial data have resulted in the ability to objectively assess the built environment [11,39,41-44]. Such objective measures are necessary to more precisely identify aspects of the built environment associated with physical activity. Second, while some studies have selected sites based on socio-economic data, few studies have selected areas based on built environment characteristics [25,26,44]. Selecting areas with a range of built environment characteristics is important to assess the relationship between the built environment and physical activity [9,16,25,42,45]. If study areas have a very similar or a highly skewed range of built environment characteristics then statistical estimates will be inaccurate [45].

This study examines the influence of the built environment on walking for transportation to work or school, walking for errands, walking for leisure and moderate physical activity among a sample of residents in suburban Metro Vancouver, Canada. This study is important because it examines a range of physical activity measures, incorporates study areas based on both built and socio-economic characteristics and objectively assesses the built environment using GIS. It adds to the growing body of international research examining the influence of the built environment on physical activity.

## Methods

### Study area

The study area includes eight neighbourhood areas with contrasting income levels and built environments across suburban municipalities of Metro Vancouver. Census tracts are small and relatively stable geographic units with an average population of 2500 to 8000 and were used as a base to select neighbourhood areas [46]. The median family income of census tracts was obtained from the 2001 Census of Canada. Census tract residential density was calculated as population per hectares of residential land. Residential land was obtained from Greater Vancouver Land Use Data which assigns a land use code to all parcels [47]. Neighbourhood areas were created by joining three to four census tracts to achieve a population between 11,000 and 17,000. Potential areas were selected based on deciles of income and residential density. Areas with the highest and lowest deciles of income were excluded as well as the lowest deciles of residential density which represented rural areas. Because our study areas are suburban and characterized by relatively low densities, the highest decile of residential density was not excluded. Among eligible census tract clusters we selected eight neighbourhood areas. Four neighbourhood areas were selected with higher residential density, two had higher median family income ($53,000-$77,000 CDN) and two had lower median family income ($32,000-$44,000). Four neighbourhood areas with lower residential density were selected, two had higher median family income ($53,000-$77,000 CDN) and two had lower median family income ($32,000-$44,000). Overall, we selected eight neighbourhood areas, two of each income/density classification (e.g. higher income, lower density). Further information on neighbourhood selection is available elsewhere [41].

### Individual data

A telephone survey was conducted by a contracted firm to obtain individual data for respondents in the selected neighbourhoods. Households were selected using Random Digit Dialling (RDD) based on a sampling frame obtained from a local telephone provider. Invalid and ineligible numbers were removed. Once a household was selected a minimum of five call-backs were attempted to minimize non-response bias. Interviews were conducted in English by experienced telephone interviewers using Computer Assisted Telephone Interviewing. The survey was conducted in February 2006, following a pilot survey in January 2006, and achieved a response rate of 29%. Data was obtained for 1935 adults aged 19 and over but 333 were excluded due to an invalid/missing postal code (n = 43) or item non-response (n = 290) resulting in a final sample of 1602. Ethics approval for data collection and analysis was sought and granted by the Office of Research Services at Simon Fraser University (application approval #38955).

### Dependent variables

Survey questions assessed participation in various types of walking and moderate physical activity. Dichotomous categories were constructed to create indicators of low physical activity and moderate or greater physical activity. Walking to work/school was assessed using the item: "In a typical week in the past 3 months how many hours did you usually spend walking to and from work or school?" Walking for errands was assessed using the item: "In a typical week in the past few months how many hours did you spend walking from home to grocery stores, banks, or to do other errands?" The responses to these questions (none, less than 1 hour, from 1 to 5 hours, from 6 to 10 hours, from 11 to 20 hours, greater than 20) were dichotomized for analysis purposes (less than one hour, one hour or greater). Moderate physical activity was assessed using the item: "In a typical week in the past 3 months how many days did you do at least 30 minutes of moderate physical activity such as brisk walking running swimming or team sports? never, 1 day, 2 or 3 days, 4 or 5 days, 6 or more". Responses were dichotomized for analysis (one day or less, two days or more). Walking for leisure was assessed from the question: "On a typical day in the past 3 months, how much time did you spend walking for leisure? 0 minutes, 15 minutes or less, 16-30 minutes, 31 minutes to one hour, over an hour." Responses were dichotomized for the analysis (15 minutes or less, greater than 15 minutes).

Individual level predictor variables include gender, age, household income, marital status, chronic conditions and obesity. Three categories of household income were constructed based on respondent self-report: low income (less than $40,000 CDN) middle income ($40,000 to $80,000 CDN) and high income ($80,000 CDN and over). Marital status of respondents was categorized as single, married/common law and divorced/widowed. Because chronic conditions may limit respondent's ability to engage in some physical activities, a variable indicating presence of a self-reported chronic condition was included. Obesity may be associated with lower levels of physical activity and to account for this, individuals with a Body Mass Index (BMI, weight (kg)/height (m) [2]) ≥ 30 were categorized as obese based on international standards [48]. BMI was based on self-reported heights and weights.

### Land use and neighbourhood income data

Line-based road network buffers were used to construct measures of land use based on prior work demonstrating that they offer a better representation than circular or "crow-fly" buffers of the neighbourhood that is accessible by walking [41]. By being constrained to the road network, as actual pedestrians are, network buffers provide a more accurate assessment of the built environment as experienced by a resident walking through each neighbourhood. This is especially true in suburban areas which typically have lower street connectivity than urban areas. Respondents were geocoded using the Statistics Canada Postal Code Conversion File which assigns a latitude and longitude co-ordinate to each respondent's self-reported postal code [49]. The British Columbia Road Network file was used to construct a one-km buffer around each postal code constrained to the road network. A 50-metre buffer was then placed around the line-based buffer to create a final buffer that was one-kilometre along the road and 50-metres on either side of the road. A detailed description of the construction of the buffer construction is available elsewhere [41].

Land use measures were constructed for each respondent's network buffer using Greater Vancouver Land Use Data. While land use codes differed across municipalities of the study region, a simplified layer has been constructed from more detailed land use codes to facilitate analysis across the region and a full description is available elsewhere [47]. Four land use categories are employed for the present analysis:

*Recreational and park land *includes parks, play grounds, fields, and trails/wooded areas.

*Residential land *includes all private and rental dwellings such as high rises, low rises, garden/town homes, and single detached homes.

*Commercial land *includes businesses with retail sales and services and professional offices.

*Institutional land *includes public offices, hospitals, libraries, community centres, schools, city hall, and correction facilities.

For each respondent's line-based road network buffer, the proportion of land for each of the four land use categories was calculated. For each category, the proportions across all respondents were divided into three tertiles (low, mid, high) and the middle category was used as the reference for analysis. A fifth standard measure of land use mix was constructed by calculating the distribution of the four land uses and the measure was divided into thirds [17].

Because neighbourhoods were selected on the basis of income, dichotomous variables were included to indicate if a neighbourhood was in the higher or lower category. This strategy has been used in other studies [26]. While density was also used as a selection variable, it was not included in analyses due to multicollinearity with the land use measures.

### Statistical analysis

The main analytical strategy used was logistic regression to predict the influence of individual and land use characteristics on the four physical activity measures. Four sets of models are presented. Each set assesses the influence of the five land use measures on a single physical activity outcome while controlling for individual predictors and neighbourhood income. All statistical analyses were conducted using SPSS version 15.0.

## Results

### Sample characteristics

Table 1 presents the characteristics of respondents: 70% spent less than one hour per week walking to work or school and 50% spent less than one hour walking for errands. For measures assessing recreational physical activity, 36% of respondents engaged in moderate physical activity once per week or less while 31% of respondents spend less than 15 minutes per day walking for leisure. The average age of respondents was 47 years and there were more female than male respondents. Chronic conditions were reported by 37% of respondents and the majority of respondents were married or common law at 64%. The proportion of respondents falling within the low- and mid-income categories was greater than the highest income category. Based on a BMI ≥ 30, 16% of respondents were classified as obese.

**Table 1:** Descriptive statistics for sample

Variable (N = 1602)	Percent	Average (SD*)
**Outcome**		

Walk to work or school less than one hour	70.22%	

Walk for errands less than one hour per week	49.75%	-

Moderate physical activity one or less days per week	36.39%	

Walk for leisure 15 minutes or less per day	31.40%	

**Predictors**		

Age	-	47.03 (14.16)

Gender (Female)	61.80%	-

Chronic conditions	37.33%	

*Marital Status*		

Single	18.66%	-

Married/Common Law	64.36%	-

Divorced or widowed	16.98%	-

*Income*		

Low (Less than $40,000)	34.64%	-

Mid ($40,000-$80,000)	38.90%	-

High (More than $80,000)	26.46%	-

Obese (BMI ≥ 30)	16.17%	

Neighbourhood: high income	50.68%	

*SD = Standard Deviation

Source: Survey of residents in eight neighbourhoods in Metro Vancouver, 2006

Table 2 presents the physical activity responses for terciles of each of the five land use measures. A gradient is evident in which each lower tercile of commercial land and land use mix had fewer respondents walking to work or school (i.e. walking less than one hour per week). For land use mix, in the highest tercile 40% of respondents do not walk for errands (i.e. walk less than one hour per week) compared to 65% in the lowest tercile. Similar results are evident for commercial land. The percent of respondents reporting low moderate physical activity (one day or less per week) is associated with higher terciles of recreational and park land, commercial land and land use mix. Walking for leisure is associated with residential land, institutional land and land use mix. For residential land, 27% of respondents in the lowest tercile do not walk for leisure (i.e. walk 15 minutes or less per day) compared to 34% in the highest tercile. Respondents living in the highest tercile of institutional land have a higher prevalence of walking for leisure than those in middle and low terciles.

**Table 2:** Physical activity outcome variables by land use thirds

		Walking to work or school	Walking for errands	Moderate physical activity	Walking for leisure
		
		Less than one hour per week	Less than one hour per week	One day or less per week	15 minutes or less per day
**Land Use**		**%**	**%**	**%**	**%**

**Recreational & park land**	Low	72.25	58.19	31.79	32.18
	
	Mid	69.85	45.51	37.45	29.96
	
	High	68.67	45.9	39.71	32.06

**Residential land**	Low	71.8	43.78	36.36	27.46
	
	Mid	67.34	45.69	40.18	32.66
	
	High	71.62	60.23	32.43	34.17

**Commercial land**	Low	74.47	64.11	32.05	32.25
	
	Mid	69.8	46.78	37.2	32.04
	
	High	66.54	38.85	39.78	29.93

**Institutional land**	Low	72.45	52.83	37.36	33.4
	
	Mid	67.04	48.59	38.61	33.71
	
	High	71.16	47.87	33.27	27.17

**Land use mix**	Low	74.41	65.23	34.38	35.55
	
	Mid	66.3	44.32	36.26	29.3
	
	High	70.02	39.85	38.55	29.61

Source: Survey of residents in eight neighbourhoods in Metro Vancouver, 2006

### Logistic regression models

Table 3 presents results of the logistic regression models. Results for 'walking to work or school - less than one hour per week' for recreational and park land are in section A. Females are more likely to walk to work or school than males. Having a chronic condition decreases the odds of walking to work or school. Respondents reporting low income are more likely to walk to school or work. The odds ratios for the individual predictors are similar across all models. None of the land use measures are significant at the *p *< 0.05 level.

**Table 3:** Logistic regression models predicting the influence of land use characteristics on physical activity

Predictors	Recreational & park land		Residential land		Commercial land		Institutional land		Land use mix	
	
	OR	**95% C.I**.	OR	**95% C.I**.	OR	**95% C.I**.	OR	**95% C.I**.	OR	**95% C.I**.
**A. Walking to work or school - less than one hour per week**										

**Age**	1.03*	(1.02,1.04)	1.03*	(1.02,1.04)	1.03*	(1.02,1.04)	1.04*	(1.03,1.05)	1.03*	(1.02,1.04)

**Female**	0.76*	(0.60,0.96)	0.76*	(0.60,0.96)	0.76*	(0.60,0.96)	0.76	(0.60,0.97)	0.75	(0.59,0.96)

**Chronic conditions**	1.36*	(1.06,1.75)	1.36*	(1.05,1.74)	1.37*	(1.07,1.76)	1.35	(1.05,1.74)	1.36	(1.05,1.74)

**Obese**	0.98	(0.72,1.33)	0.98	(0.72,1.33)	0.99	(0.72,1.35)	0.99	(0.72,1.34)	0.99	(0.73,1.35)

**Family Income**										

**Low**	0.68*	(0.52,0.90)	0.68*	(0.51,0.90)	0.68*	(0.52,0.90)	0.66	(0.50,0.88)	0.69*	(0.52,0.91)

**Mid (ref)**										

**High**	1.30	(0.97,1.74)	1.29	(0.96,1.73)	1.27	(0.95,1.71)	1.29	(0.96,1.74)	1.28	(0.95,1.72)

**Marital status**										

**Single**	1.18	(0.87,1.62)	1.17	(0.86,1.60)	1.19	(0.87,1.62)	1.20	(0.88,1.64)	1.21	(0.88,1.65)

**Married (ref)**	1.00		1.00		1.00		1.00		1.00	

**Divorced**	1.57*	(1.08,2.28)	1.53*	(1.05,2.23)	1.55*	(1.06,2.25)	1.54*	(1.06,2.23)	1.57*	(1.08,2.28)

**Neighbourhood income**										

**Higher**	1.22	(0.97,1.55)	1.23	(0.97,1.57)	1.14	(0.88,1.48)	1.25	(0.98,1.59)	1.16	(0.91,1.49)

**Land use**										

**Low**	1.25	(0.95,1.65)	0.96	(0.72,1.28)	1.28	(0.92,1.77)	1.23	(0.92,1.63)	1.29	(0.97,1.72)

**Mid**	1.12	(0.85,1.47)	0.95	(0.72,1.25)	1.01	(0.76,1.36)	0.90	(0.68,1.19)	0.96	(0.73,1.27)

**High (ref)**	1.00		1.00		1.00		1.00		1.00	

**B. Walking for errands - less than one hour per week**										

**Age**	1.00	(1.00,1.01)	1.00	(1.00,1.01)	1.00	(1.00,1.01)	1.00	(1.00,1.01)	1.01	(1.00,1.01)

**Female**	1.02	(0.83,1.26)	1.00	(0.81,1.24)	1.00	(0.81,1.24)	1.03	(0.84,1.27)	1.01	(0.81,1.24)

**Chronic conditions**	0.89	(0.71,1.10)	0.91	(0.73,1.14)	0.91	(0.73,1.14)	0.88	(0.70,1.09)	0.90	(0.72,1.13)

**Obese**	1.22	(0.93,1.61)	1.24	(0.94,1.63)	1.27	(0.97,1.68)	1.24	(0.95,1.63)	1.30	(0.98,1.71)

**Family Income**										

**Low**	0.72*	(0.56,0.92)	0.72*	(0.56,0.92)	0.72*	(0.56,0.93)	0.69*	(0.54,0.89)	0.71*	(0.55,0.92)

**Mid (ref)**										

**High**	1.14	(0.88,1.47)	1.06	(0.82,1.38)	1.07	(0.82,1.39)	1.12	(0.86,1.45)	1.06	(0.82,1.38)

**Marital status**										

**Single**	0.89	(0.66,1.19)	0.87	(0.65,1.17)	0.91	(0.68,1.22)	0.87	(0.65,1.16)	0.95	(0.70,1.27)

**Married (ref)**	1.00		1.00		1.00		1.00		1.00	

**Divorced**	1.08	(0.79,1.46)	1.11	(0.81,1.51)	1.10	(0.81,1.50)	1.04	(0.77,1.42)	1.17	(0.85,1.60)

**Neighbourhood income**										

**Higher**	1.53*	(1.24,1.89)	1.64*	(1.32,2.03)	1.12	(0.89,1.42)	1.62*	(1.31,2.02)	1.39*	(1.12,1.74)

**Land use**										

**Low**	1.53*	(1.19,1.96)	0.49*	(0.38,0.63)	2.48*	(1.85,3.31)	1.42*	(1.10,1.82)	2.65*	(2.04,3.43)

**Mid**	0.93	(0.72,1.18)	0.62*	(0.48,0.8)	1.27	(0.97,1.65)	1.18	(0.91,1.52)	1.34*	(1.04,1.72)

**High (ref)**	1.00		1.00		1.00		1.00		1.00	

**C. Moderate physical activity - one day or less per week**										

**Age**	1.01	(1.00,1.02)	1.01*	(1.00,1.02)	1.01	(1.00,1.02)	1.01	(1.00,1.02)	1.01	(1.00,1.02)

**Female**	0.73*	(0.58,0.91)	0.73*	(0.59,0.91)	0.73*	(0.58,0.91)	0.73*	(0.59,0.91)	0.73*	(0.58,0.91)

**Chronic conditions**	1.36*	(1.08,1.71)	1.36*	(1.08,1.71)	1.37*	(1.09,1.72)	1.37*	(1.09,1.72)	1.37*	(1.09,1.72)

**Obese**	1.69*	(1.29,2.23)	1.68*	(1.28,2.21)	1.69*	(1.28,2.23)	1.69*	(1.29,2.23)	1.7*	(1.29,2.23)

**Family Income**										

**Low**	1.33*	(1.03,1.72)	1.34*	(1.03,1.73)	1.35*	(1.04,1.75)	1.34*	(1.03,1.74)	1.36*	(1.05,1.76)

**Mid (ref)**										

**High**	0.59*	(0.44,0.78)	0.59*	(0.45,0.79)	0.59*	(0.44,0.78)	0.58*	(0.43,0.77)	0.58*	(0.44,0.77)

**Marital status**										

**Single**	0.73*	(0.53,0.99)	0.74*	(0.54,1.00)	0.74	(0.55,1.01)	0.73*	(0.54,1.00)	0.75	(0.55,1.02)

**Married (ref)**										

**Divorced**	1.02	(0.75,1.40)	1.04	(0.76,1.42)	1.05	(0.77,1.43)	1.04	(0.76,1.43)	1.04	(0.76,1.43)

**Neighbourhood income**										

**Higher**	0.87	(0.70,1.09)	0.89	(0.71,1.12)	0.85	(0.67,1.09)	0.92	(0.73,1.16)	0.85	(0.67,1.07)

**Land use**										

**Low**	0.81	(0.62,1.05)	1.05	(0.81,1.37)	1.06	(0.78,1.43)	1.17	(0.89,1.52)	1.08	(0.83,1.41)

**Mid**	1.01	(0.78,1.30)	1.20	(0.92,1.56)	1.07	(0.81,1.40)	1.26	(0.97,1.65)	0.92	(0.71,1.20)

**High (ref)**	1.00		1.00		1.00		1.00		1.00	

**D. Walking for leisure - 15 minutes or less per day**										

**Age**	1.00	(0.99,1.01)	1.00	(0.99,1.01)	1.00	(0.99,1.01)	1.00	(0.99,1.01)	1.00	(1.00,1.01)

**Female**	0.68*	(0.54,0.85)	0.67*	(0.54,0.84)	0.68*	(0.54,0.84)	0.68*	(0.55,0.86)	0.67*	(0.54,0.84)

**Chronic conditions**	1.08	(0.85,1.36)	1.10	(0.87,1.39)	1.08	(0.86,1.37)	1.08	(0.85,1.36)	1.06	(0.84,1.35)

**Obese**	1.33*	(1.01,1.76)	1.33*	(1.00,1.76)	1.34*	(1.01,1.77)	1.34*	(1.01,1.77)	1.36*	(1.02,1.80)

**Family Income**										

**Low**	1.19	(0.91,1.56)	1.20	(0.91,1.57)	1.20	(0.92,1.58)	1.18	(0.90,1.55)	1.22	(0.93,1.60)

**Mid (ref)**										

**High**	1.09	(0.82,1.43)	1.06	(0.80,1.40)	1.08	(0.82,1.42)	1.06	(0.80,1.40)	1.07	(0.81,1.41)

**Marital status**										

**Single**	1.05	(0.77,1.43)	1.06	(0.77,1.44)	1.06	(0.78,1.44)	1.04	(0.77,1.42)	1.09	(0.8,1.48)

**Married (ref)**										

**Divorced**	0.90	(0.65,1.25)	0.93	(0.66,1.29)	0.91	(0.65,1.27)	0.90	(0.65,1.25)	0.94	(0.67,1.30)

**Neighbourhood income**										

**Higher**	1.07	(0.85,1.34)	1.13	(0.90,1.43)	0.99	(0.77,1.27)	1.14	(0.91,1.44)	1.01	(0.80,1.28)

**Land use**										

**Low**	1.03	(0.79,1.34)	0.70*	(0.54,0.92)	1.21	(0.89,1.64)	1.30	(0.99,1.71)	1.36*	(1.04,1.78)

**Mid**	0.94	(0.72,1.22)	0.93	(0.72,1.21)	1.14	(0.86,1.52)	1.32*	(1.00,1.73)	0.99	(0.75,1.30)

**High (ref)**	1.00		1.00		1.00		1.00		1.00	

Source: Survey of residents in eight neighbourhoods in Metro Vancouver, 2006

*Significant at the *P *< 0.05 level.

Section B of Table 3 presents the results for 'walking for errands - less than one hour per week'. Few individual level predictor variables are significant. For all models, age, gender, chronic conditions and obesity are not associated with walking for errands. Across all land use models, those in a low income family are more likely to walk for errands. Living in a high income neighbourhood increases the odds of not walking for errands. Living in the lowest tercile of recreational and park land increases the odds of not walking for errands (OR 1.53, 95% CI 1.19 to 1.96). Relative to living in the highest tercile of residential land, respondents living in middle and low terciles are more likely to walk for errands. Respondents living in the lowest tercile of commercial land use are less likely to walk for errands (OR 2.48, 95%CI 1.85 to 3.31). Living in a low or middle tercile of land use mix increases the odds of not walking for errands relative to living in the highest tercile and the odds ratios show a stepwise pattern.

Section C of Table 3 presents the results for moderate physical activity. Several individual level predictors are significant and the odds ratios are similar across models. Being female increases the odds of participating in moderate physical activity while having a chronic condition and being obese increases the odds of not participating in moderate physical activity. Relative to being from a middle income family, being from a low income family increases the odds of not participating in moderate physical activity and being from a high income family decreases the odds of not participating. None of the land use variables are significantly associated with moderate physical activity.

The model results for walking for leisure are presented in section D of Table 3. Only two individual predictor variables are significant across all five models. Being female reduces the odds of not walking for leisure and being obese increases the odds of not walking for leisure. Chronic conditions are not associated with walking for leisure. Living in the lowest tercile of residential land reduces the odds of not walking for leisure (OR 0.70, 95% CI 0.54 to 0.92) relative to living in the highest tercile. Living in low or middle terciles of institutional land increases the odds of not walking for leisure but the p-value for the lowest tercile is marginally significant (*p *= 0.06). Relative to living in the highest tercile of land use mix, living in the lowest tercile increases the odds of not walking for leisure (OR 1.36, 95% CI 1.04, 1.78). Recreational and park land was not associated with walking for leisure.

## Discussion

The primary purpose of this paper is to assess the influence of land use on various types of physical activity among a sample of suburban residents. In our study we evaluated four specific dimensions, walking for errands, walking to work or school, walking for leisure and moderate physical activity. Walking for errands was associated with increasing commercial land, institutional land and land use mix which corresponds to several other studies finding that measures of land use mix or proximity to destinations are associated with walking for transport [9,26,50,51]. These results suggest that residents in close proximity to commercial services and/or to public institutions are more physically active. These findings underscore the importance of the built environment in shaping participation in physical activity.

This study also found that residents living in the lowest tercile of park and recreational land were less likely to report low levels of walking for errands. This is in contrast to another study finding that measures of the natural environment were not associated with walking for transport [52]. It is possible that residents use parks and green ways to walk for errands. However, it is unclear why park and recreational land is associated with walking for errands but not walking for leisure.

Logistic regression models did not find significant difference in walking to work/school by land use terciles (commercial and land use mix). This finding is in contrast to other studies showing that land use mix is associated with increased walking for commuting [53]. This result may be explained by the fact that the study areas are suburban and major places of employment or school may not be in close proximity. For example, in a suburban municipality in our study region, Coquitlam, only 37% of employed individuals actually work in the City of Coquitlam [54].

The logistic regression models did not find a significant difference in moderate physical activity by land use terciles and similar results have been reported elsewhere [26,36]. The presence of recreational or park land was not associated with walking for leisure or moderate physical activity and other studies have also reported a lack of association [36,52]. The measure used simply assessed the presence of recreational and park land and it is possible that this measure is not sufficient to show significant associations. Data detailing the specific type of park and recreational land (e.g. beach, playground, foot paths) as well as aesthetics or quality may be needed to demonstrate associations [55].

Walking for leisure was associated with residential land, institutional land and land use mix. The results indicate that individuals living in areas with low land use mix and low institutional land are less likely to walk for leisure.

There are several strengths to this study. The study design included neighbourhoods with a range of residential densities, which is considered to be important in examining how the built environment influences physical activity and overcomes limitations of other studies [45]. Another strength is that network buffers were used which may better assess salient aspects of the built environment as experienced by pedestrians [41]. In this study we were able to assess the influence of the built environment on four dimensions of physical activity. This study has several limitations as well. The survey item assessing walking for leisure did not specify walking from home. Respondents may not necessarily walk for leisure in their immediate neighbourhood. In this study one-kilometre network buffers were used and it is possible that a differing buffer size may be more appropriate for some individuals (e.g. seniors) or for different types of physical activity (e.g. running, walking to grocery store). Following previous studies we adjusted for obesity, however other studies have found that obesity is independently related to aspects of the built environment [56]. Measures of physical activity and obesity were obtained using self-reports which have limitations compared to direct measures [57]. The survey was administered in February and the rainy and cold weather experienced in the study region during this time of year may mean that the rates of physical activity are more conservative than if the survey was conducted in a warmer month. Seasonal differences may impact certain types of physical activity more than others. However, a strength of this study is that all participants were assessed within a short period of time minimizing differences between respondents due to seasonal variation in weather. In this study we did not assess aesthetics or and social dimensions such as safety, cohesions and trust which may influence physical activity [7]. While models were adjusted for neighbourhood income there were not enough neighbourhoods to conduct multilevel analysis [58].

## Conclusions

This study adds to the growing body of research examining the influence of the built environment on physical activity. In contrast to previous studies, this study included a range of physical activity variables, assessed areas with a range of built environments and measured land use using high resolution spatial data. This study found that walking for errands showed greater association with the neighbourhood environment than other dimensions of physical activity. Walking for leisure was associated with institutional land and land use mix and indicates that access to public institutions such as community centres and libraries may promote physical activity. Recreation and park land was not associated with walking for leisure or moderate physical activity. Future research should use more refined measures of recreational and park land (e.g. play ground, foot path) as well as measures of quality and aesthetics. The findings of this research demonstrate that the built environment can influence physical activity though the strength of the relationship depends on the type of physical activity considered.

## Competing interests

The authors declare that they have no competing interests.

## Authors' contributions

LNO conceived of the project and prepared the manuscript. NCS developed the initial GIS methodology and AWH provided a novel adaptation of the GIS methodology. NCS and AWH assisted with preparing the manuscript. MH was involved with the design and implementation of the survey and edited the manuscript. All authors read and approved the final manuscript

## Note

The responsibility for the content of the paper rests solely with the author, and should not be attributed to the institutions which the authors are affiliated.

## Pre-publication history

The pre-publication history for this paper can be accessed here:

http://www.biomedcentral.com/1471-2458/11/959/prepub
